# Lead poisoning; a neglected potential diagnosis in abdominal pain

**DOI:** 10.1186/s12876-020-01284-1

**Published:** 2020-05-06

**Authors:** Mahtab Shabani, Seyed Kaveh Hadeiy, Parinaz Parhizgar, Nasim Zamani, Hamid Mehrad, Hossein Hassanian-Moghaddam, Scott Phillips

**Affiliations:** 1 Private Gastroentrologist, Tehran, Iran; 2 Social Determinants of Health Research Center, Shahid Beheshti University of Medical Sciences, Tehran, Iran; 3 Department of Clinical Toxicology, Loghman Hakim Hospital, Shahid Beheshti University of Medical Sciences, Tehran, Iran; 4 University of Colorado Anchutz Medical Campus, Rocky Mountain Poison & Drug Safety, Denver, CO and Washington Poison Center, Seattle, WA USA

**Keywords:** Opium, Addiction, Lead, Poisoning, Abdominal pain, Gastrointestinal symptoms

## Abstract

**Background:**

Abdominal pain may be a presenting symptom of lead poisoning and is often difficult to diagnose. This study aimed to determine the prevalence of abdominal pain in patients seen in the Laghman Hakim Hospital ED and GI clinic who were lead-intoxicated, with or without opiate use disorder.

**Methods:**

Between July 2017 and January 2018, patients seen in the ED and GI clinic of Loghman Hakim Hospital with unexplained abdominal pain or abdominal pain resistant to treatment were enrolled. Informed consent was obtained from potential enrollees. For standardization, a pre-designed data collection tool was developed for uniform data acquisition. Opiate use was determined historically. For this study, lead poisoning was defined as a blood lead level (BLL) greater than or equal to 30 μg/dL (1.45 μmol/L) with concomitant GI symptoms.

**Results:**

Of 125 patients admitted, 28 (22.4%) had BLLs higher than 30 μg/dL. None of the patients had signs and symptoms of opioid withdrawal syndrome during evaluation. Elevated BLLs were significantly correlated with oral opium use/abuse, history of addiction for over the preceding 12 years. The daily opium use was more than 2.75 g. There was a statistical correlation between lead toxicity and abdominal pain consistency and intensity, constipation, and paresthesias. Anemia, leukocytosis, and abnormal liver enzyme tests were laboratory findings associated with lead toxicity. Four patients died, one of whom was diagnosed with lead toxicity.

**Conclusion:**

Lead toxicity should be considered in the potential differential diagnosis of severe and resistant abdominal pain in patients referring to general EDs or GI clinics if a positive history of opium abuse exists.

## Background

Acute abdominal pain is one of the leading reasons for seeking medical care in emergency and outpatient departments [1]. Abdominal pain has a diverse diagnostic schema, which ranges from mild self-limiting conditions to life-threatening emergencies. It is believed 20–40% of abdominal pain etiologies remain unknown at the time of discharge [2]. Lead toxicity is on the differential diagnosis for abdominal pain, albiet more often of a chronic nature. The United States Centers for Disease Control and Prevention (CDC) suggests that blood lead levels (BLLs) of greater than 5 mg/dL are elevated.

During the difficult time of an endemic or epidemic, premature closure of a diagnosis is exacerbated by anchoring, the tendency for clinicians to stick with the initial impression even as new information becomes available. Because of the broad differential diagnosis of abdominal pain, misdiagnosis may be minimized by a formalized approach. Anchoring is exacerbated during times such as the opium endemic in Iran. In order to avoid this diagnostic misadventure, clinicians must keep an open mind as to the diagnosis and use the iterative process of the differential diagnosis. By following a predetermined algorithm, diagnostic errors can be minimized.

Lead poisoning may occur from many situations, including occupational exposure (e.g., battery manufacturing workers, lead solder workers, automobile radiator repair, etc.) [3], tile workers [4], and potters [5] or paint chips [6]. Lead-based paints before 1950, old pipes, house dusts, imported toys, and herbal and Ayurvedic medications are among the possible sources for lead poisoning [7]. Lead poisoning is generally asymptomatic; however, it can present with abdominal pain, constipation, anemia, hearing loss, fatigue, neuropathy and neurotoxicity, encephalopathy, renal diseases, abortions, osteopenia, and even death.

Treatment of lead poisoning is centered on prevention, eliminating lead exposure, proper diet (rich in calcium and iron), education, and if indicated, treatment using chelating agents including (dimercaprol (BAL), calcium disodium ethylenediaminetetraacetic acid (CaNa2 EDTA), succimer (DMSA,2–3 mesodimercaptosuccinic acid), and D-penicillamine) [8, 9].

Prevalence of lead poisoning has dropped over the recent years because of the lead safety and preventive measures and programs [10] although epidemics of lead poisoning due to adulterated opium or herbal medicine were recently reported worldwide [11, 12].

In 2016, an epidemic of lead poisoning occurred in Iran due to consumption of lead-contaminated opium. Lead was added to opium in increase the weight of the product and income. Some estimates of the size of the outbreak to involve more than 40,000 people [11]. Abdominal pain is a symptom of lead poisoning [12–15], which has historically resulted in unnecessary diagnostic and even therapeutic procedures. Some may undergo unnecessary surgery to seek for possible intra-abdominal pathologies because the theaters (mostly gastroenterologists and surgeons) may not be familiar with lead-contaminated opium in their patients [16].

Patients presenting to our emergency department (ED) and gastrointestinal (GI) diseases clinic for abdominal pain were evaluated to determine the frequency of lead poisoning as an etiological cause. Both inpatients and those who were managed as outpatients were evaluated. A major focus of this study was to examine possible factors correlating with lead poisoning. A listing of signs and symptoms noted in this cohort with abdominal pain were recorded that may also be related to lead poisoning.

## Methods

A prospective, non-randomized, observational study was conducted between July 2017 and January 2018. Patients evaluated in the emergency and GI outpatient clinic with the chief complaint of abdominal pain of unknown origin were enrolled into the study.

### Inclusion criteria

1. All genders
2. Age > 18 years
3. Generalize abdominal pain
4. Duration of Pain > 7 days

### Exclusion criteria

1. Fever
2. Shock
3. EKG changes suggestive of ACS
4. Acute onset of abdominal pain
5. Peritoneal signs
6. Trauma

**Fig. 1:**
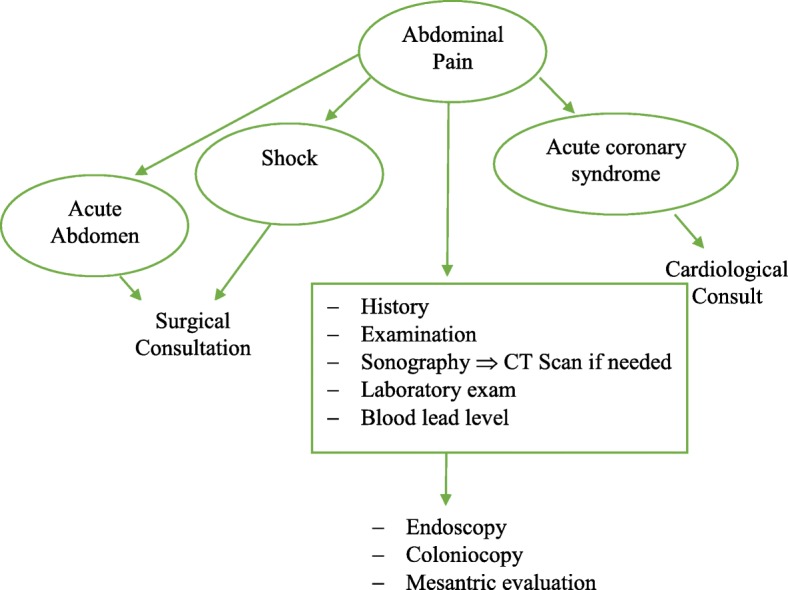
Algorythm enrollment of the patients

An algorhythmic approach was used (see Fig. 1) for study enrolment.

All surgical and cardiovascular causes of abdominal pain were ruled out using approach to abdominal pain published by UpToDate® [17]. Patients meeting the inclusion criteria were further evaluated for the possibility of lead toxicity.

At the time of this study, our region was experiencing a lead endemic from contaminated opium. Because of the endemic, lead colic, while normally uncommon, had a heightened sense of urgency for diagnosis. Patients were considered as being lead-poisoned if their BLL was more than 30 μg/dL in conjunction with abdominal pain.

Patients were informed of study details and voluntarily participated after providing informed consent. A predetermined study questionnaire was designed for this study. In the questionnaire, the patients were asked about their demographics, the quality/ location/ time of initiation and severity of their abdominal pain, previous history of abdominal pain and any given treatment or diagnostic procedures performed for pain management, prescriptive medication use, particular eating habit, herbal medicine consumption, previous history of lead toxicity, history of addiction, route and daily amount of the opium use, duration of addiction, and on-presentation signs and symptoms other than abdominal pain. Presenting laboratory testing, primary and final diagnoses, treatments performed, duration of hospitalization, need for ICU admission, and standard questions determining whether the patient is a user of opium (based on ICD criteria) including their history of substance abuse, gradual increase of drug consumption, unsuccessful tries to quit drug consumption, possible damaged social life because of drug addiction, excessive eagerness for taking drug, continuing use of drug even after being aware of its side effects, presence of withdrawal signs during its discontinuation, etc. were also asked and recorded. Laboratory tests including blood biochemistry and liver function tests were also collected.

Capillary blood lead levels were checked by Lead Care II device using voltametry method. This device determines the BLLs between 3.2 to 65 μg/dL [18]. The levels below and above these limits are determined as “low” and “high” levels. For those with “low” results, a mean of 1.6 μg/dL was determined to be used in data analysis. For any level above 65 μg/dL, the number 65 was inserted into the statistical package for social sciences (SPSS) file (version 24) and data was analyzed.

Quantitative variables were described based on their pattern of distribution, normal and non-normal, with mean and median in addition to their interquartile range and minimum to maximum ranges. Kolmogorov-Smirnov test was conducted on the quantitative variables to determine their distribution (normal or non-normal). Continuous variables were compared between two groups of patients with normal and abnormal BLLs using t-test and Mann-Whitney U Test. For the factors that showed significant correlations, cut-off points with highest amounts of sensitivity and specificity were determined by using ROC Curve test. Evaluation of categorical variables was done by conducting Chi square test. *P* values less than 0.05 were considered to be statistically significant. 95% confidence intervals and odds ratios were demonstrated for showing the power of association. Multivariable analysis was conducted by defining logistic regression models.

### Sample size

Considering the previously performed pilot study on 93 patients which showed an average frequency of BLLs higher than 10 μg/dL to be about 47.3% [18], the desired 95% level of confidence, and the desired ±1.8 μg/dL accepted margin of error, a sample size of 142 was determined. Sixty-seven patients were expected to have BLLs higher than 10 μg/dL.

## Results

Data on one hundred twenty-five patients was collected. Of the 125 cases, 62 (49.6%) were seen in the ED and 63 (50.4%) were seen in outpatient GI clinic. Almost 80% of the patients were male. When the final diagnosis of lead poisoning was confirmed, 27 out of 28 patients were men. Twenty-eight patients (22.4%) had BLLs higher than 30 μg/dL and 64 and 108 patients had BLLs above 10 μg/dL and 5 μg/dL, respectively.

Four cases died, one of whom was diagnosed with lead poisoning and had died due to aspiration pneumonia. Other causes of death were gastrointestinal bleeding, opioid overdose, and congestive heart failure. In 83 (66.4%) patients, there was a positive history of opium dependence. All 28 patients with confirmed lead poisoning had the history of addiction and 15 (53.8%) received chelating agents. Demographic data and lab test results are summarized in Tables 1 and 2. In the patients with high BLL, four patients underwent invasive procedures (colonoscopy and endoscopy) to rule out severe background causes of abdominal pain. None of our patients underwent a surgery. Table 3 illustrates the cofactors which showed significant correlation with lead poisoning. In the multivariate analysis, among the factors that showed univariate correlation with elevated BLL with few missing data, only constipation (OR 8.9 CI 95% 1.3–59.3) and leukocytosis (OR 12.6 CI 95% 1.7–95.6) were significantly related to high BLLs. Multivariate regression information has been summarized in Table 4.

**Table 1:** Patients’ demographic characteristics (*n* = 125)

Age; mean (range)	48 ± 16.9 (16 to 85)
Gender (M/F)	100/25
Inpatient/outpatient	62/63
Addiction history	83 (66.4%) with positive addiction history
Treatment	16 received chelating agents (12.8%), 62 (49.6%) were treated conservatively, 2 underwent surgery (1.6%), 45 missing (36%)
Outcome (death/recovery)	4/121

**Table 2:** On-presentation lab tests

	Patients with BLL ≤ 30 mg/dL *N* = 97	Patients with BLL ≥ 30 mg/dL *N* = 28	*P* value	Total *N* = 125
Median [IQR] WBC (mcL) min-max	7‚800 [6,700, 9,375]	12‚100 [8,800, 13,700]	<0.001	8‚400 [6,800, 10,200]
	3‚200–23,000	5‚100–25,800		3‚200–25,800
Median [IQR] HCT (%)	39 [36.9, 42.1]	35.10 [29.3, 38.8]	0.001	38.50 [35.1, 42.0]
min-max	10.0–50.7	22.5–44.2		10.0–50.7
Median [IQR] RBC (milion/mm^3^)	4.43 [3.95, 4.90]	4.16 [3.32, 4.50]	0.016	4.33 [3.90, 4.85]
min-max	1.10–6.29	2.60–5.32		1.10–6.29
Median [IQR] Hgb (g/dL)	13.8 [12.1, 14.7]	11.70 [9.6, 13.0]	0.000	13.0 [11.7, 14.3]
min-max	3.0–17.2	7.4–15.2		3.0–17.2
Median MCV (fL)	87.0 [84.0, 89.0]	87 [82.5, 92.0]	0.756	87 [83.0, 89.9]
min-max	61.5–116.0	77–95.71		61.50–116
Median LDH (U/L)	396.0 [327.5, 560.0]	462.0 [414.5, 610.2]	0.023	415.0 [353.0, 581.0]
min-max	30.0–2108.0	299.0–890.0		30.0–2108.0
Median PLT (10^9^/L)	201.5 [176.5, 242.0]	242 [185.2, 306.0]	0.013	209.5 [179.2, 248.7]
min-max	61.0–540.0	98.0–435.0		61.0–540.0
Median AST (U/L)	20.0 [14.0, 30.0]	34.0 [123.2, 60.5]	<0.001	22.0 [16.0, 35.0]
min-max	10.0–469.0	12.0–197.0		10.0–469.0
Median ALT (U/L)	19.0 [15.5, 27.0]	36.0 [19.2, 68.0]	0.003	21.0 [15.0, 37.0]
min-max	10.0–1122.0	12.0–219.0		10.0–1122.0
Median BS (mg/dL)	103 [91, 123]	123 [111, 168]	0.001	109 [94, 136]
min-max	49–368	95–367		49–367
Median BUN (mg/dL)	28.5 [20.0, 40.8]	30.0 [23.0, 43.0]	0.342	30.0 [20.0, 42.0]
min-max	10.0–204.0	12.5–131.0		10.0–204.0
Median Cr (mg/dL)	1.1 [0.9, 1.2]	11 [1.0, 1.1]	0.495	1.1 [0.9, 1.2]
min-max	0.7–3.4	0.7–1.3		0.7–3.4
Median Na (mEq/L)	139.5 [138.0, 142.0]	137.0 [135.0, 140.0]	0.038	139.0 [137.0, 141.0]
min-max	125.0–145.0	130.0–148.0		125.0–148.0
Mean (SD) K (mEq/L)	4.0 (0.4)	4.1 (0.4)	0.295	4.1 (0.4)
min–max	3.7–4.3	4.0–4.3		3.8–4.3
Median [IQR] ESR (mm/h)	6.0 [2.0,15.0]	21.0 [8.5, 45.5]	0.034	8.5 [3.2, 28.7]
min-max	1.0–51.0	3.0–86.0		1.0–86.0
Median [IQR] CRP (mg/L)	7.0 [3.0, 41.0]	6.8 [1.4, 77.2]	0.934	7.0 [2.7, 43.0]
min-max	0.2–155	0.2–96.5		0.2–155.0
Median [IQR] Amylase (U/L)	62 [42, 76]	53 [31, 91]	0.405	58 [36, 79]
min-max	11–293	16–102		11–293
Median [IQR] Lipase (U/L)	29 [20, 37]	21 [20, 37]	0.406	28 [20, 37]
min-max	16–103	9–114		9–114
Median [IQR] Troponine (ng/mL)	0.0015 [0.0002]	0.0010 [0.0010, 0.1027]	0.815	0.0010 [0.0010, 0.0160]
min-max	0.000–1.500	0.000–0.385		0.000–1.500
Median [IQR] CPK (U/L)	86 [52, 177]	137 [63, 366]	0.205	93 [60, 225]
min-max	2.5–3758	35–2742		2.5–3758
Median [IQR] CK MB (U/L)	15 [12, 22]	18 [10, 24]	0.980	15 [11, 22]
min-max	6–73	10–80		6–80
Mean (SD) pH (VBG)	7.41 (0.07)	7.44 (0.07)	0.093	7.42 (0.07)
min-max	7.37–7.47	7.40–7.50		7.37–7.47
Mean (SD) HCO_3_ (mEq/L,VBG)	24.9 (4.2)	26.5 (4.7)	0.550	25.1 (4.4)
(min–max)	22.6–27.9	21.9–28.1		22.6–28.0
Mean (SD) PCO_2_ (mEq/LVBG)	39.6 (10.7)	37.3 (9.4)	0.550	38.9 (10.3)
min-max	31.8–44.5	32.6–44.0		31.8–44.0
Median [IQR] PT (seconds)	13.1 [12.5, 13.7)	12.2 [12.0, 13.9]	0.110	12.9 [12.2, 13.6]
min-max	10.9–27.0	10.5–19.3		10.5–27.0
Median [IQR] PTT (seconds)	31.0 [28.0, 34.4]	30.0 [26.9, 37.9]	0.823	31.0 [27.1, 34.6]
min-max	24.2–45.4	26.1–60.0		24.0–60.0
Median [IQR] INR	1.0 [0.8, 3.5]	1.0 [0.8, 2.0]	0.680	1.0 [0.9, 1.2]
median (min-max)	0.83–3.50	0.83–2.00		0.83–3.50

**Table 3:** Factors with significant association with BLLs above 30 μg/dL

	*P* value	Odd ratio	Confidence interval 95% ( upper-lower)	
Addiction history	<0.001	1.428	1.276	1.721
Gender (male/female)	0.014	0.113	0.015	0.874
Pain type (colic pain- constant pain)	0.024	2.744	1.119	6.724
Pain duration (LESS OR MORE THAN A WEEK) 64 sensitivity 64 specificity	0.010	3.278	1.302	8.250
Ingestion of opium ( as the way for taking drug)	0.001	16.059	2.026	1.721
Duration of addiction (more than 12 years)	0.039	2.720	1.037	7.132
Abnormal AST test	0.003	4.911	1.636	14.743
Abnormal ALT test	0.000	6.875	2.309	20.468
Leukocytosis More than 9050	<0.001	8.095	3.043	21.536
RBC Count Less than 4.2759 sensitivity 64 specificity	0.042	2.547	1.017	6.379
HematocritLess than 37.172 sen 64 spec	0.001	4.622	1.809	11.808
Platelet countMore than 21973 sensitivity 64 specificity	0.001	4.819	1.822	12.729
ESRMore than 1177 sensitivity 67 specificity	0.02	7.000	1.200	40.828
LDHMore than 41283 sensitivity 61 specificity	0.002	7.813	1.947	31.346
NaLess than 137.577 sensitivity 52 specificity	0.011	3.584	1.301	9.876
Blood sugarMore than 112.574 sensitivity 65 specificity	0.002	5.051	1.747	14.601
Anemia	<0.001	6.253	2.175	17.975
Constipation	<0.001	7.464	2.320	20.487
Paresthesia	0.002	7.455	1.725	32.215
Continuous using of opium even with being aware of its side effects	0.031	5.031	1.041	24.317
Excessive eagerness to use opium	0.038	3.694	1.091	12.510
Daily amount of opium intake (more than 2.75 gr)	0.005	5.257	1.577	17.526

**Table 4:** Multivariate regression

	*P* value	OR	CI 95 % (upper – lower)	
Addiction history	.998	295607633.200	.000	.
Gender	.809	.645	.018	22.773
Pain type (colic/constant)	.656	1.458	.278	7.652
Pain Duration	.496	1.760	.346	8.955
leukocytosis	.014	12.609	1.662	95.633
RBC count	.979	1.029	.113	9.345
HCT	.942	.910	.071	11.636
PLT	.277	2.775	.441	17.472
Anemia	.111	7.386	.632	86.280
Constipation	.024	8.885	1.330	59.374
Paresthesia	.097	8.611	.680	109.082

## Discussion

In this study, all 28 patients with confirmed diagnosis of lead poisoning also had a history of opium addiction and opiate use disorder (OUD). Although some patients were polysubstance users, all poisoned patients had the history of OUD. There was no significant correlation between consumption of herbal medicines, eating habits, and occupation and lead poisoning as many of our patients had jobs that did not predispose them to lead exposure. This suggests adulterated opium as the main source of lead poisoning. Patients who took daily amounts of more than 2.75 g of opium had a 5.2-time greater risk of development of lead poisoning. There was also a correlation between lead poisoning and OUD in this study. Persons continued to abuse opium after being aware of its consequences although this correlation was not statistically significant. This may be due to the fact that this innate feeling will result in taking greater amounts of opium. A correlation between the duration of opium addiction and lead poisoning was evident.

Before analysis, it was presumed that patients’ dependence on opium and their tendency to continue opium even after being aware of its consequences would correlate with daily amount of opium intake. However, statistical analysis did not show an association. This may be due to an inaccurate history of daily opium intake.

Significant difference in gender and lead poisoning in this study was found. In this cohort, 83 patients with positive addiction history were make, 78 males and five females. This may demand further evaluation of role of gender in development of lead poisoning.

In our study, there was a significant correlation between lead poisoning and lab tests. In the poisoned group, anemia (Hgb < 13 mg/dL) was 6.3 times more common compared to the normal group. Simultaneous presence of lead poisoning and anemia has also been mentioned in the previous studies. Lead interferes with the production of heme [18–21]. In fact, during the heme production, two pivotal enzymes (5-aminolevulinic acid dehydratase and ferrochelatase) are inhibited [22, 23]. There was also a connection between BLL and RBC count which is similar to the results withdrawn by previous authors [22, 23]. Terayama suggested that lead exposure would result in cell membrane changes that reduced the lifetime of erythrocytes [23]. In addition, lead is linked with lower level of erythropoietin [24] both of which mechanisms can cause a reduction in RBC count. Decreased amounts of erythropoietin, decreased RBC life span, and deformability besides the decrease in heme production justify the correlation of decreased hematocrit and higher BLLs.

There was also an association between platelet (PLT) count and BLL. In some previous studies, BLL was associated with decreased platelet count [25] while in some others, increased PLT number has been mentioned [26]. However, complete blood count (CBC) profile of our patients demonstrated a higher PLT count in lead-poisoned patients. It has also been mentioned that the risk of stroke is higher in the patients with abnormal BLL [27]. Higher PLT numbers in these patients can be a potential explanation for this finding.

WBC count more than 9050 per mm^3^ had an 8.1-time higher frequency of lead poisoning. Similar findings have been reported by Katavolos et al. in 2007 [26]; however, they considered leukocytosis as an accidental finding. Paucity of studies in this field warrants further studies to elucidate the association between higher BLL and leukocytosis.

As Mirzaei et al. declared in 2018 [27], paresthesia was associated with lead poisoning. Presence of paresthesia is due to the neuropathic effects of lead. Typically, lead firstly involves the motor nerves of wrists and finger extensors and then spreads to other muscles [28]. Several explanations have been given for neuropathic effects of lead. Impaired Ca2+ hemostasis due to lead exposure will result in nerve apoptosis [29]. Lead also disrupts the fluid normal hemostasis in neurons which results in high cellular pressure and consequent segmental demyelination in neurons [30].

A significant correlation between constipation and lead poisoning was reveled in this study. As mentioned earlier, there were statistical correlations between lead poisoning and pain consistency and severity. Lead can cause abdominal pain through several mechanisms. Lead will negatively affect the motility of intestines [31]. It can be due to the effect of lead on intestinal neural web or on smooth muscles. It can also be due to the increase in δ aminolevulinic acid (a porphyrin precursor) as a result of induced porphyrinopathy by lead poisoning [32]. There is also evidence for the role of lead in acute pancreatitis. Lead also interferes with sodium transporter channels of the intestine. These mechanisms explain the causality of lead in abdominal pain but the question that can be raised here is how can lead toxicity be differed from opium withdrawal syndrome or narcotic bowel syndrome especially regarding to the fact that all of the cases in this study were due to adulterated opium. Characteristics of opioids withdrawal syndrome (OWS) and narcotic bowel syndrome (NBS) is listed in Table 5 [33, 34]. Since the main concern of this was to focus on the presence of lead poisoning in individual seeking medical care with unknown or treatment resistant abdominal pain, patients that clearly showed such conditions were not enrolled.

**Table 5:** Characteristics of opioid withdrawal syndrome (OWS) and narcotic bowel syndrome (NBS)

OWS	lacrimation or rhinorrhea, piloerection “goose flesh,” myalgia, diarrhea, nausea/vomiting, pupillary dilation and photophobia, insomnia, autonomic hyperactivity (tachypnea, hyperreflexia, tachycardia, sweating, hypertension, hyperthermia), and yawning.
NBS	constipation, nausea, bloating, ileus and predominantly abdominal pain

Another prominent factor which significantly correlated with lead poisoning was abnormal liver enzyme tests possibly due to oxidative/antioxidant cycle disturbances in the liver tissue [35] or depletion of the antioxidants savings of the cells [36]. Three out of 21 patients who had primarily unjustified abdominal pain and three of those 24 who were diagnosed primarily with GI problems were lead-poisoned. Paying attention to lead poisoning as a potential diagnosis could rule out the need for invasive procedures like colonoscopy and endoscopy. Although none of our lead-poisoned patients had undergone surgery before the diagnosis, the possibility of this diagnosis during handling acute abdomen should always been borne in mind.

## Conclusion

Lead poisoning should be considered as a potential diagnosis in patients with positive history of opium or ayurvedic medicine intake; however, differential diagnosis of lead poisoning in abdominal pain should be correlated with local epidemiologic data on lead poisoning.

### Limitations

Our study was conducted at the time of the outbreak of lead poisoning due to adulterated opium. This will explain relatively high numbers of patient with lead toxicity. Almost half of our cases were patients with limited access to follow up after their discharge and this issue caused relatively high amount of missing data especially in laboratory findings in our study. Some patients did not even return for treatment causing almost 45 missing data in treatment information. It is possible the patients discontinued using opium but this is another limitation of this study. Another aim was to determine the districts in Tehran with most prevalence of dealing with adulterated opium with lead; however, because of the paucity of cases this could not be done.

## Data Availability

The data generated by and used in the study is available from the corresponding author upon reasonable request.

## Abbreviations

BLL: Blood lead level
CBC: Complete blood count
CI: Confidence interwall
ED: Emergency Department
GI: Gastrointestinal
ICD: International Classification of Disease
ICU: Intensive care unit
NBS: Narcotic bowel syndrome
OR: Odds ratio
OUD: Opiate use disorder
OWS: Opioids withdrawal syndrome
PLT: Platelet
SPSS: Statistical Package for Social Sciences
