# Correction to: Lead poisoning; a neglected potential diagnosis in abdominal pain

**DOI:** 10.1186/s12876-021-01920-4

**Published:** 2021-10-29

**Authors:** Mahtab Shabani, Seyed Kaveh Hadeiy, Parinaz Parhizgar, Nasim Zamani, Hamid Mehrad, Hossein Hassanian-Moghaddam, Scott Phillips

**Affiliations:** 1 Private Gastroentrologist, Tehran, Iran; 2 Social Determinants of Health Research Center, Shahid Beheshti University of Medical Sciences, Tehran, Iran; 3 Department of Clinical Toxicology, Loghman Hakim Hospital, Shahid Beheshti University of Medical Sciences, Tehran, Iran; 4 Department of Internal Medicine, Loghman Hakim Educational Hospital, School of Medicine, Shahid Beheshti University of Medical Sciences, Tehran, Iran; 5 University of Colorado Anchutz Medical Campus, Rocky Mountain Poison & Drug Safety, Denver, CO and Washington Poison Center, Seattle, WA USA

## Correction to: BMC Gastroenterology 20, 134 (2020) 10.1186/s12876-020-01284-1

In this article the affiliation details for Hamid Mehrad were incorrectly given as ‘Private Gastroentrologist, Tehran, Iran' but should have been ‘Department of Internal Medicine, Loghman Hakim Educational Hospital, School of Medicine, Shahid Beheshti University of Medical Sciences, Tehran, Iran'.

The original article [1] has been updated.
